# Host Serine Proteases TMPRSS2 and TMPRSS11D Mediate Proteolytic Activation and Trypsin-Independent Infection in Group A Rotaviruses

**DOI:** 10.1128/JVI.00398-21

**Published:** 2021-05-10

**Authors:** Michihito Sasaki, Yukari Itakura, Mai Kishimoto, Koshiro Tabata, Kentaro Uemura, Naoto Ito, Makoto Sugiyama, Christida E. Wastika, Yasuko Orba, Hirofumi Sawa

**Affiliations:** a Division of Molecular Pathobiology, Research Center for Zoonosis Control, Hokkaido University, Sapporo, Japan; b Drug Discovery and Disease Research Laboratory, Shionogi & Co., Ltd., Osaka, Japan; c Laboratory of Biomolecular Science, Faculty of Pharmaceutical Sciences, Hokkaido University, Sapporo, Japan; d Laboratory of Zoonotic Diseases, Faculty of Applied Biological Sciences, Gifu University, Gifu, Japan; e Global Institution for Collaborative Research and Education (GI-CoRE), Hokkaido University, Sapporo, Japan; f Global Virus Network, Baltimore, Maryland, USA; Instituto de Biotecnologia/UNAM

**Keywords:** group A rotavirus, proteolytic activation, type II transmembrane serine proteases, VP4, viral entry

## Abstract

Group A rotaviruses (RVAs) are representative enteric virus species and major causes of diarrhea in humans and animals. The RVA virion is a triple-layered particle, and the outermost layer consists of the glycoprotein VP7 and spike protein VP4. To increase the infectivity of RVA, VP4 is proteolytically cleaved into VP5* and VP8* subunits by trypsin; these subunits form a rigid spike structure on the virion surface. In this study, we investigated the growth of RVAs in cells transduced with type II transmembrane serine proteases (TTSPs), which cleave fusion proteins and promote infection by respiratory viruses, such as influenza viruses, paramyxoviruses, and coronaviruses. We identified TMPRSS2 and TMPRSS11D as host TTSPs that mediate trypsin-independent and multicycle infection by human and animal RVA strains. *In vitro* cleavage assays revealed that recombinant TMPRSS11D cleaved RVA VP4. We also found that TMPRSS2 and TMPRSS11D promote the infectious entry of immature RVA virions, but they could not activate nascent progeny virions in the late phase of infection. This observation differed from the TTSP-mediated activation process of paramyxoviruses, revealing the existence of virus species-specific activation processes in TTSPs. Our study provides new insights into the interaction between RVAs and host factors, and TTSP-transduced cells offer potential advantages for RVA research and development.

**IMPORTANCE** Proteolytic cleavage of the viral VP4 protein is essential for virion maturation and infectivity in group A rotaviruses (RVAs). In cell culture, RVAs are propagated in culture medium supplemented with the exogenous protease trypsin, which cleaves VP4 and induces the maturation of progeny RVA virions. In this study, we demonstrated that the host proteases TMPRSS2 and TMPRSS11D mediate the trypsin-independent infection and growth of RVAs. Our data revealed that the proteolytic activation of RVAs by TMPRSS2 and TMPRSS11D occurs at the viral entry step. Because TMPRSS2 and TMPRSS11D gene expression induced similar or higher levels of RVA growth as trypsin-supplemented culture, this approach offers potential advantages for RVA research and development.

## Abstract (precis)

Proteolytic cleavage of the viral VP4 protein is essential for virion maturation and infectivity in group A rotaviruses (RVAs). In cell culture, RVAs are propagated in culture medium supplemented with the exogenous protease trypsin, which cleaves VP4 and induces the maturation of progeny RVA virions.

## INTRODUCTION

Membrane-anchored serine proteases participate in various cellular physiological processes and contribute to the development and homeostasis of cells (1). Type II transmembrane serine proteases (TTSPs) consist of an N-terminal cytosolic domain, a single-pass transmembrane domain, and a C-terminal extracellular serine protease domain that carries the conserved catalytic triad of serine, aspartate, and histidine (1). Previous studies demonstrated that TTSPs cleave viral fusion proteins and facilitate protease-dependent infection by viruses, including influenza viruses, paramyxoviruses, coronaviruses, and mammalian orthoreoviruses (2–12). TMPRSS2 is expressed in the respiratory epithelium, and it is a well-studied member of the TTSP family with respect to the role of TTSPs in infection by respiratory viruses (13–15). Extensive research revealed that TMPRSS2 mediates the proteolytic cleavage of viral fusion proteins and virion maturation at early (viral entry) and/or late (virion assembly) steps, thereby facilitating viral spread *in vitro* and *in vivo* (14–20). In addition to TMPRSS2, it has been reported that other TTSPs, such as TMPRSS11D (also known as HAT), TMPRSS11E (known as DESC1), and TMPRSS13 (known as MSPL), also mediate the proteolytic activation of influenza viruses and coronaviruses, whereas their roles in infection *in vivo* remain to be elucidated (3, 11, 12).

Group A rotaviruses (RVAs) are the major causative agents of diarrhea in humans and animals. RVA infection causes more than 120,000 diarrheal deaths globally each year (21). Two oral live attenuated vaccines (Rotarix and RotaTeq) have been licensed since 2006, and they are currently available in 98 countries (22) (http://rotacouncil.org/vaccine-introduction/global-introduction-status/). The RVA virion is a nonenveloped and triple-layered particle with an outermost layer that consists of the glycoprotein VP7 and spike protein VP4. Exogenous proteases cleave VP4 (88 kDa in size) into the subunits VP5* (60 kDa) and VP8* (28 kDa), which remain noncovalently bound (23). The proteolytic cleavage of VP4 forms a rigid spike structure on the RVA virion, and it is essential for the acquisition of virus infectivity (24, 25). For RVA propagation in cell culture, RVA-inoculated cells are cultured in serum-free medium supplemented with trypsin, which cleaves VP4 and enables multicycle infection by RVA. Little is known regarding the association between host TTSPs and RVA infection.

Given the strong dependence of the activation and growth of RVA on exogenous proteases, host TTSPs may have the potential to activate RVAs and facilitate their infectivity. In this study, we explored this hypothesis and demonstrated that host TTSPs mediate trypsin-independent infection by RVAs. We identified TMPRSS2 and TMPRSS11D as TTSPs that facilitate infection by human and animal RVA strains in culture without trypsin supplementation. In the latter part of this study, we investigated the mechanism of trypsin-independent RVA infection and demonstrated that TMPRSS2 and TMPRSS11D activate immature RVA virions at the viral entry phase.

## RESULTS

### Multicycle infection by the simian RVA SA11 in TTSP-transduced cells.

**FIG 1:**
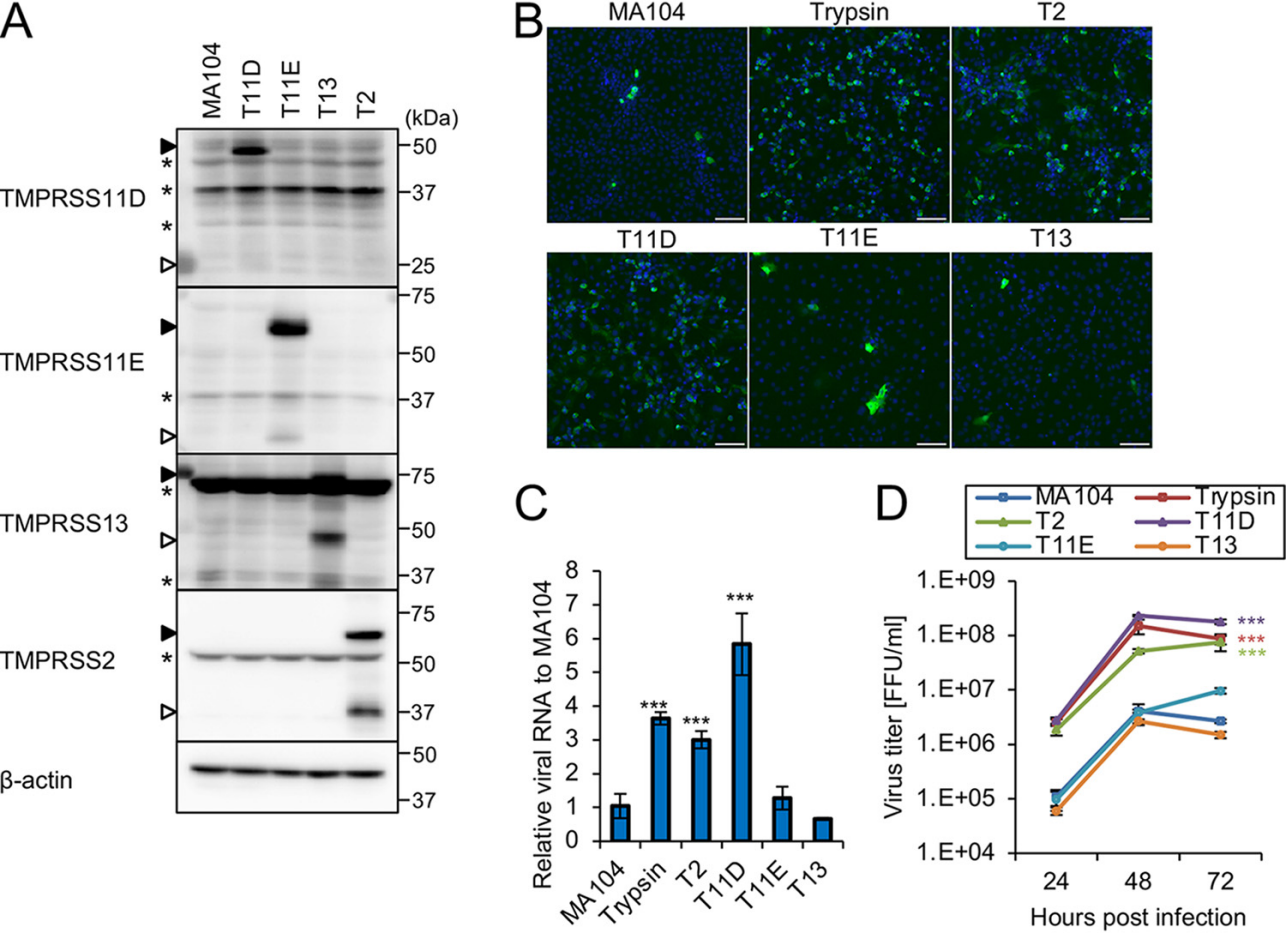
Infection and growth of the group A rotavirus (RVA) SA11 strain in cells expressing exogenous type II transmembrane serine proteases (TTSPs). (A) Generation of MA104 cells transduced with lentiviral vectors expressing TMPRSS2 (T2), TMPRSS11D (T11D), TMPRSS11E (T11E), or TMPRSS13 (T13). Expression of TTSPs was confirmed by Western blotting with antibodies specific to the proteins indicated on the left. The full-length and cleaved forms are indicated by closed and open arrowheads, respectively. Asterisks indicate nonspecific bands that cross-reacted with antibodies. (B to D) The RVA SA11 strain grown in the absence of trypsin was inoculated into MA104 or TTSP-expressing cells (T2, T11D, T11E, and T13 cells) and cultured without trypsin for 48 h. MA104 cells infected with SA11 and cultured with trypsin (shown as trypsin) were used as a positive control. (B) Cells were stained with anti-RVA antibody (green) and Hoechst 33342 nuclear dye (blue) at 48 h postinfection. Bars, 100 μm. (C) Total RNA was extracted from cells and analyzed by quantitative reverse transcription (qRT)-PCR. The levels of SA11 *VP6* RNA were normalized to β-actin mRNA levels. (D) Viral titers in the cultures were determined using a focus assay. Statistical difference at 72 hpi was measured. The values in the graphs are expressed as the means ± standard deviations (SDs) from triplicate samples. One-way analysis of variance with Dunnett’s test was employed to determine statistical significance. Significant differences are indicated by asterisks: ***, *P* < 0.001.

Using a lentiviral vector system, we generated MA104 cells stably expressing human TMPRSS2 (T2 cells), human TMPRSS11D (T11D cells), human TMPRSS11E (T11E cells), or human TMPRSS13 (T13 cells). Western blot analysis revealed the expression signal for each transduced gene in stable cells but not in parental MA104 cells (Fig. 1A). Because TTSPs are processed by autocatalytic cleavage (1), both full-length and cleaved forms of TTSPs were detected in the blots, whereas the signal for the cleaved form of TMPRSS11D was faint. Additional bands were observed in all lanes, including for mock-transfected cells, presumably due to nonspecific antibody binding (Fig. 1A). SA11, an RVA strain well adapted to growth in cell culture, was activated by trypsin pretreatment and inoculated to the cells. After a single round of infection in MA104 cells in the absence of trypsin, the progeny SA11 viruses were used to infect naive cells, and they spread throughout the culture in serum-free medium supplemented with trypsin (trypsin in Fig. 1B) but not in the medium lacking trypsin (MA104 in Fig. 1B). A number of SA11-infected cells were observed in T2 and T11D cell cultures even in trypsin-free medium at 48 h postinfection (hpi) (Fig. 1B). Consistent with the results of the immunofluorescent assay, SA11 VP6 RNA levels in T2 and T11D cells were significantly higher than those in MA104 cells in trypsin-free culture (Fig. 1C). Notably, the progeny virus titers in T2 and T11D cells in trypsin-free culture were higher than those in parental MA104 cells in trypsin-free culture (*P* < 0.001; T2 and T11D versus MA104) and comparable to those in MA104 cells in trypsin-supplemented culture (shown as trypsin in Fig. 1D) at 24, 48, and 72 hpi (Fig. 1D). Conversely, the growth of SA11 in T11E and T13 cells was similar to that in MA104 cells. These results indicate that TMPRSS2 and TMPRSS11D mediate the growth of SA11 in cells without trypsin supplementation.

### Multicycle infection by human and bovine RVAs in TTSP-transduced cells.

**FIG 2:**
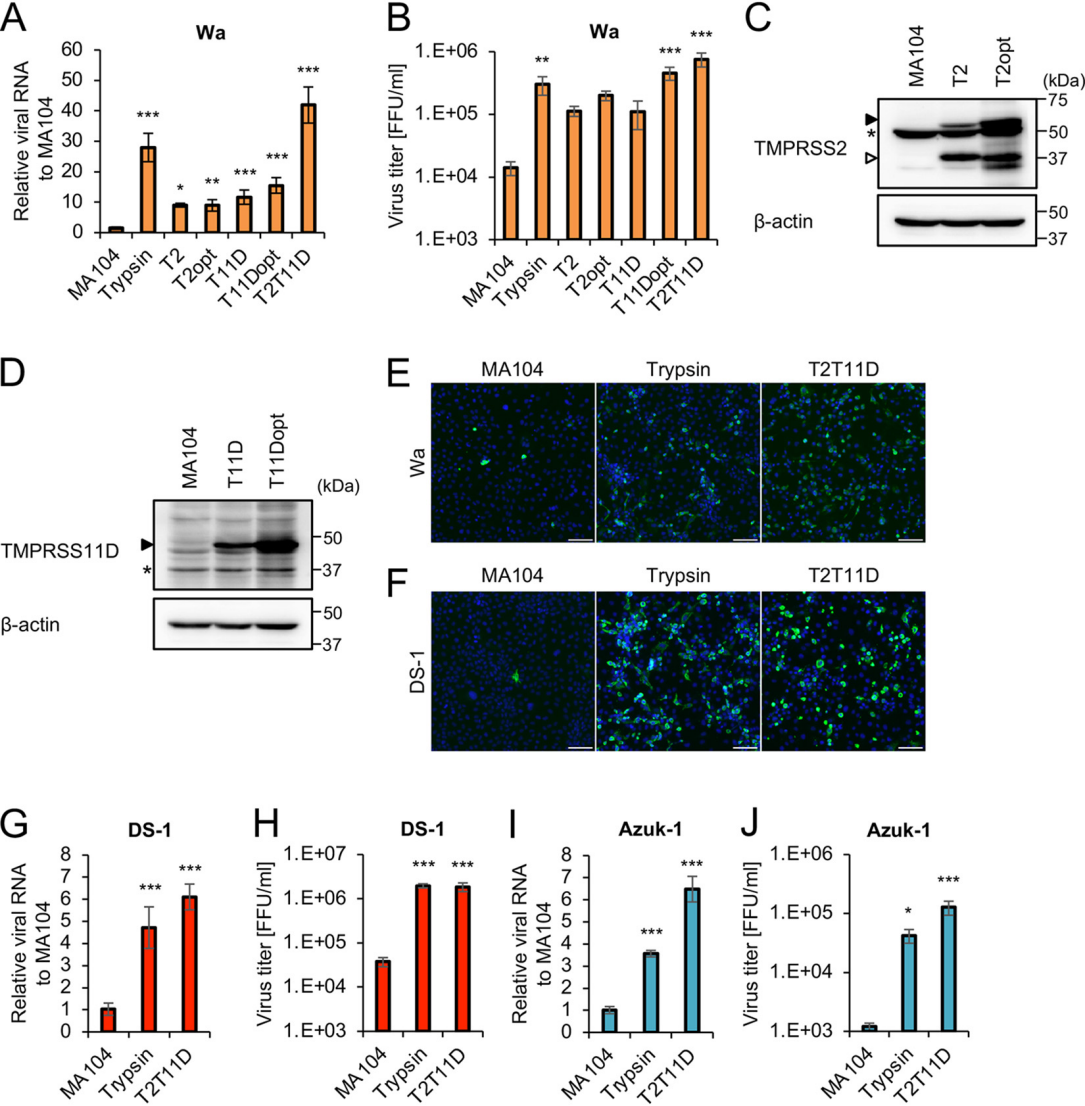
Infection and growth of human and bovine group A rotavirus (RVA) strains in cells expressing exogenous type II transmembrane serine proteases (TTSPs). (A and B) MA104 and TTSP-expressing cells were infected with the human RVA Wa strain and cultured without trypsin for 48 h. MA104 cells cultured with trypsin (shown as trypsin) were used as the positive control. (A) Total RNA was extracted from cells and analyzed via qRT-PCR. The levels of Wa *NSP3* were normalized to those of β-actin mRNA. (B) Viral titers in the cultures were determined using a focus assay. Generation of MA104 cells transduced with lentiviral vectors expressing codon-optimized TMPRSS2 (T2opt) (C) or TMPRSS11D (T11Dopt) (D). The enhanced expression of TMPRSS2 and TMPRSS11D was confirmed by Western blotting with antibodies specific to the proteins indicated on the left. The full-length and cleaved forms of TMPRSS2 are indicated by closed and open arrowheads, respectively. The asterisks indicate nonspecific bands. (E and F) Indirect immunofluorescence assay. Cells were infected with human RVA Wa (E) or human RVA DS-1 (F) and cultured without trypsin for 48 h. MA104 cells infected with each strain and cultured with trypsin were used as positive controls. Cells were stained with anti-RVA antibody (green) and Hoechst 33342 nuclear dye (blue). Bars, 100 μm. Cells were infected with human RVA DS-1 (G and H) and bovine RVA Azuk-1 (I and J) and cultured without trypsin for 48 h. MA104 cells cultured with trypsin were used as the positive control. (G and I) Total RNA was extracted from cells and analyzed via qRT-PCR. DS-1 *NSP3* and Azuk-1 *VP6* RNA levels were normalized to β-actin mRNA levels. (H and J) Viral titers in the cultures were determined using a focus assay. The values in the graphs are expressed as the means ± SDs from triplicate samples. *, *P* < 0.05; **, *P* < 0.01; ***, *P* < 0.001 by one-way analysis of variance with Dunnett’s test.

We first examined the growth of the human RVA Wa strain in T2 and T11D cells. Whereas the growth of Wa in the absence of trypsin was demonstrated via quantitative reverse transcription-PCR (qRT-PCR) and titration assays, the growth of the virus in T2 and T11D cells was lower than that in MA104 cells in trypsin-supplemented culture (Fig. 2A and B). We next attempted to enhance the expression levels of TMPRSS2 and TMPRSS11D by codon optimization of these transduced gene sequences, as it has been reported that a high concentration of protease increases the infectivity and yield of RVAs (26, 27). We generated stable MA104 cells, in which codon-optimized TMPRSS2 and TMPRSS11D were introduced, designated T2opt and T11Dopt cells, respectively (Fig. 2C and D). In addition, we generated other stable MA104 cells expressing both codon-optimized TMPRSS2 and TMPRSS11D (T2T11D cells). Thereafter, we performed a growth assay for the Wa strain using these generated cell lines. The growth of Wa in T2opt and T11Dopt cells was higher than that in T2 and T11D cells, respectively (Fig. 2A and B). Notably, the growth of Wa in T2T11D cells was higher than that in MA104 cells in trypsin-supplemented culture (Fig. 2A and B). An immunofluorescence assay using anti-RVA antibody revealed many positive signals in T2T11D cells in the absence of trypsin and in MA104 cells with trypsin (Fig. 2E), indicating that the Wa strain efficiently replicates and spreads in cultured T2T11D cells.

To validate the effectiveness of T2T11D cells to support the growth of different RVA strains, human RVA DS-1 and bovine RVA Azuk-1 strains were also examined using these assays. Experiments using these two strains were performed in a roller culture apparatus for efficient viral growth. Compared with the MA104 cells in trypsin-supplemented culture, T2T11D cells in a trypsin-free culture showed clear cytopathic effects (CPE) after infection with RVA DS-1 (Fig. 2F). Trypsin-independent viral growth was observed in T2T11D cells infected with DS-1 (Fig. 2G and H) and Azuk-1 (Fig. 2I and J). Taken together, these results indicate that exogenous expression of both TMPRSS2 and TMPRSS11D enables the trypsin-independent growth of multiple RVA strains in cells.

### Contribution of the enzymatic activities of TTSPs to RVA growth.

**FIG 3:**
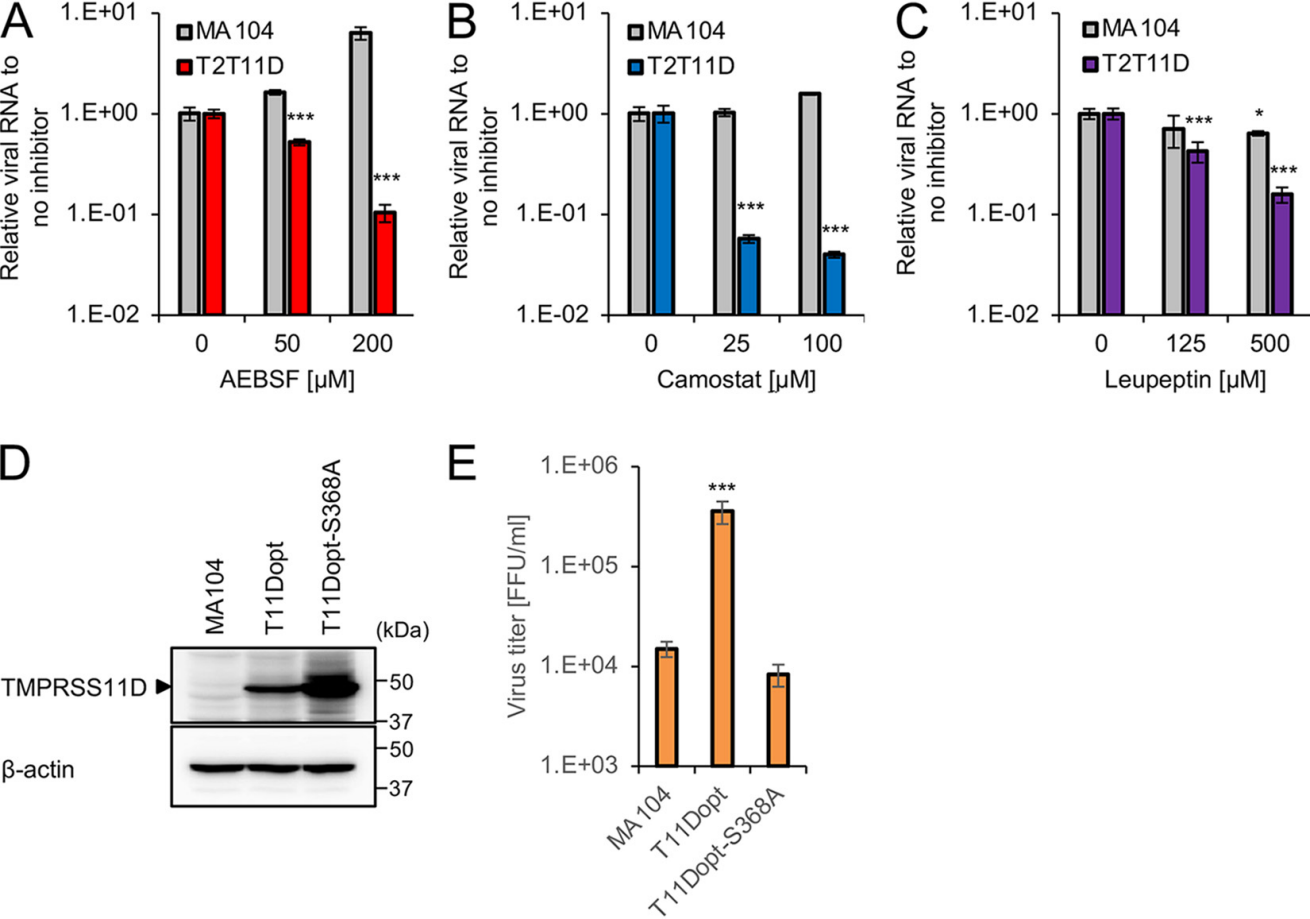
Effect of type II transmembrane serine protease (TTSP) inhibitors and proteolytic inactive mutation into TMPRSS11D on group A rotavirus (RVA) infectivity. MA104 and T2T11D cells infected with the RVA Wa strain were cultured for 48 h with the TTSP inhibitors AEBSF (A), camostat (B), and leupeptin (C). Total RNA was extracted from cells, and the relative levels of Wa *NSP3* RNA were measured via qRT-PCR. The β-actin mRNA levels were used as reference control. (D) Generation of MA104 cells transduced with a lentiviral vector expressing the protease-inactive mutant TMPRSS11D-S368A with codon optimization (T11Dopt-S368A). The expression of TMPRSS11D-S368A was confirmed by Western blotting. (E) Viral titers in the cultures were determined using a focus assay at 48 h postinfection by the RVA Wa strain. The values in the graphs are expressed as the means ± SDs from triplicate samples. *, *P* < 0.05; ***, *P* < 0.001 by one-way analysis of variance with Dunnett’s test.

To examine whether the enzymatic activities of TMPRSS2 and TMPRSS11D compensate for the lack of trypsin in culture, viral growth was monitored in the presence of the protease inhibitors 4-(2-aminoethyl) benzenesulfonyl fluoride (AEBSF), camostat, and leupeptin. These inhibitors have been used to inhibit serine proteases, including trypsin and TTSPs, and to reduce the infectivity of influenza viruses and coronaviruses (5, 9, 10, 12, 28). After inoculation of the RVA Wa strain, MA104 and T2T11D cells were cultured in the presence of inhibitors for 48 h. Virus growth was evaluated by qRT-PCR, as residual inhibitors hinder the titration assay of culture samples. All three inhibitors significantly reduced viral growth in T2T11D cells, but not in MA104 cells, in a concentration-dependent manner (Fig. 3A to C), suggesting that these inhibitors reduce RVA growth through inhibition of protease activity of TTSPs in cells.

To further investigate the effect of the protease activity of TTSPs on RVA growth, we transduced MA104 cells with protease activity-defective, codon-optimized TMPRSS11D carrying a mutation in the catalytic triad (S368A) and obtained stable MA104 cells expressing the TMPRSS11D mutant (T11Dopt-S368A cells) (29). The expression level of the TMPRSS11D mutant in T11Dopt-S368A cells was relatively higher than that of TMPRSS11D in T11Dopt cells (Fig. 3D), whereas the expression of the TMPRSS11D mutant did not enhance the progeny virus titer (Fig. 3E). These results indicate that TTSPs facilitate the growth of RVAs through their enzymatic activity.

### Application of T2T11D cells for RVA propagation in FBS-supplemented culture.

**FIG 4:**
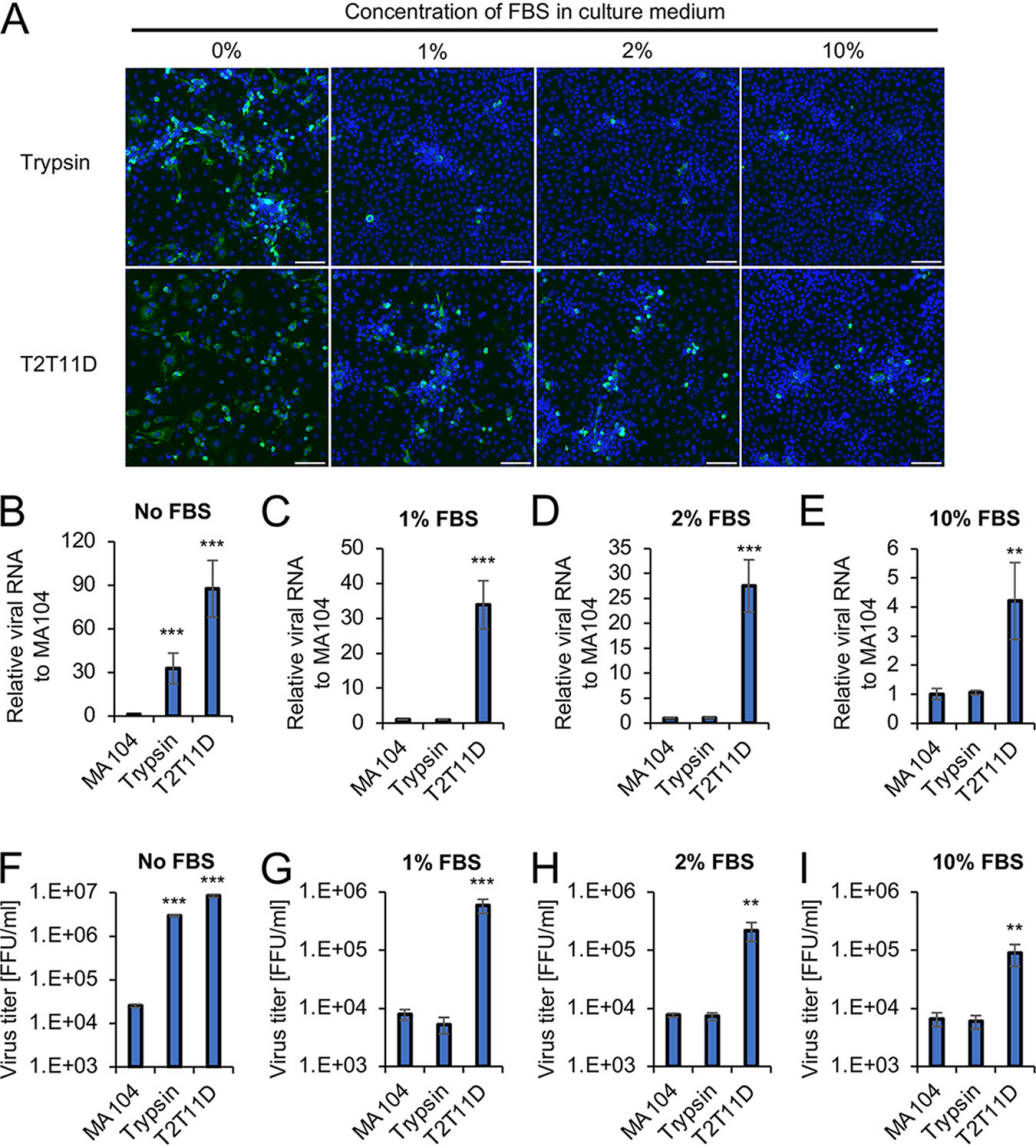
Group A rotavirus (RVA) growth in type II transmembrane serine protease (TTSP)-expressing cells in the presence of fetal bovine serum (FBS). Cells infected with the RVA Wa strain were cultured in medium containing the indicated concentrations of FBS for 48 h. (A) RVA-infected cells were detected via indirect immunofluorescence using anti-RVA antibody (green) and Hoechst 33342 (blue). Bars, 100 μm. (B to E) Total RNA was extracted from RVA-infected cells and analyzed by qRT-PCR. The levels of Wa *NSP3* RNA were normalized to β-actin mRNA levels. (F to I) Cell-associated viruses were recovered using a freeze-thaw method in fresh serum-free medium and titrated using a focus assay. The values in the graphs are expressed as the means ± SDs from triplicate samples. **, *P* < 0.01; ***, *P* < 0.001 by one-way analysis of variance with Dunnett’s test.

RVA was propagated in fetal bovine serum (FBS)-free medium, as FBS neutralizes the activity of trypsin. We tested the growth of the Wa strain in T2T11D cells in the presence of different FBS concentrations. In the presence of FBS, trypsin supplementation did not enhance the infection and spread of RVAs in MA104 cells (Fig. 4A). Notably, RVA infection spread throughout the culture of T2T11D cells, even in medium containing FBS (Fig. 4A). Viral RNA levels were higher in T2T11D cells than in MA104 cells irrespective of the FBS concentration, whereas the viral RNA level in T2T11D cells gradually decreased in an FBS concentration-dependent manner (Fig. 4B to E). After washing out culture medium, cell-associated viruses were recovered by a freeze-thaw method in fresh serum-free medium and titrated using a focus assay. Similarly, the virus titers in T2T11D cells were higher than those in MA104 cells during culture in FBS-supplemented medium (Fig. 4F to I). As expected, exogenous trypsin supplementation failed to increase RVA growth in MA104 cells cultured in the medium containing FBS (Fig. 4A to I). These findings indicated that T2T11D cells promote RVA spread and growth, even in low FBS concentrations, whereas RVA growth in T2T11D cells was impaired in high FBS concentrations, which indicated that the protease activities of TTSPs and trypsin were inhibited in the presence of FBS.

### Proteolytic cleavage of RVA VP4 by TMPRSS11D.

**FIG 5:**
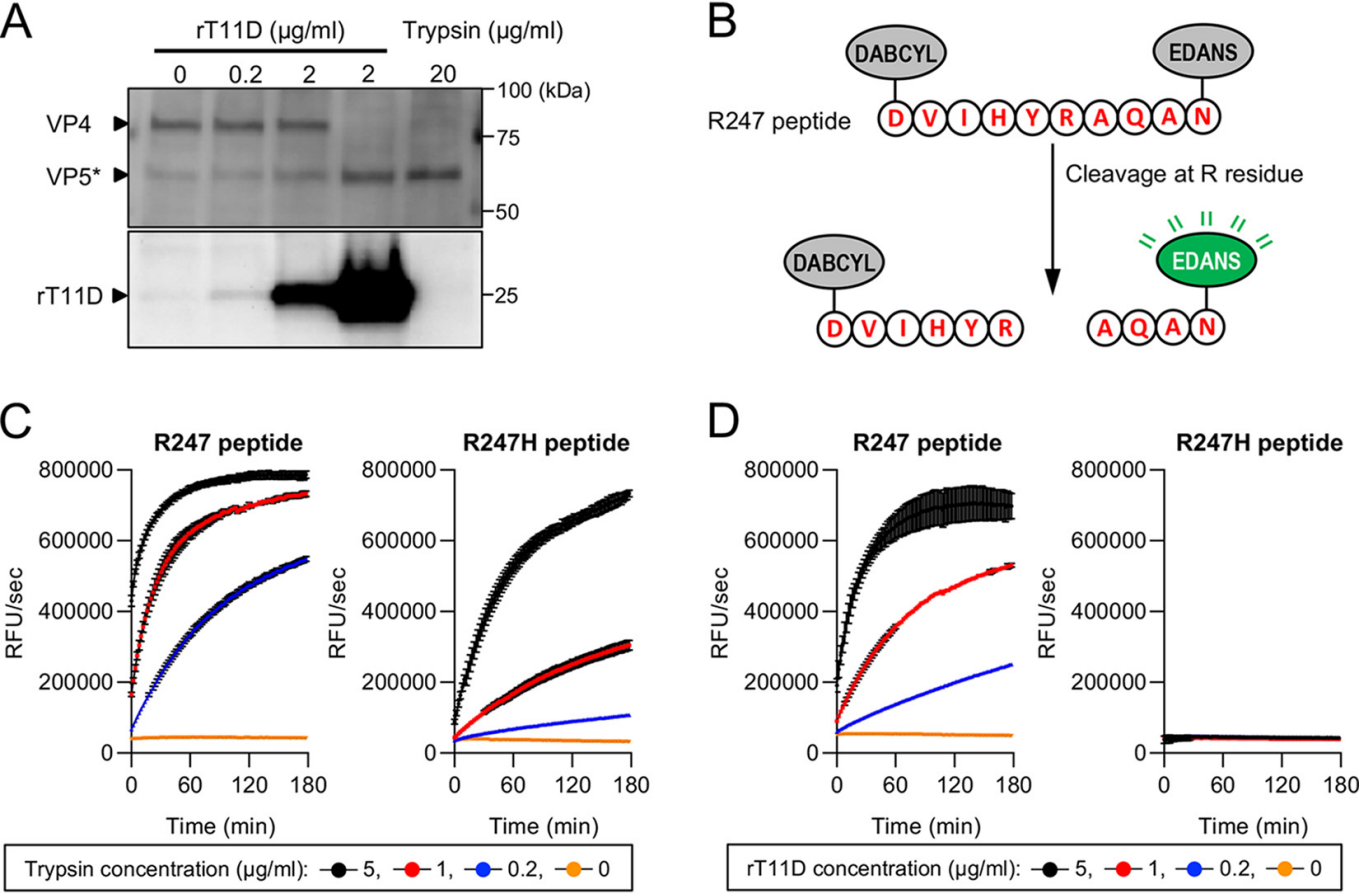
Proteolytic cleavage of VP4 by TMPRSS11D. (A) Immature SA11 virions were incubated with the indicated concentrations of recombinant TMPRSS11D (rT11D) or trypsin for 3 h. Thereafter, samples were subjected to Western blotting using anti-SA11 VP5* antibody or anti-TMPRSS11D antibody. Arrowheads indicate the signals of the indicated proteins. (B) Schematic representation of the internally quenched fluorogenic R247 peptide. The peptide sequence corresponds to the amino acid sequence between 242 and 251 of SA11. Through peptide cleavage, EDANS at the C terminus of the peptide was released from the quencher DABCYL at the N terminus and emitted a fluorescence signal. (C and D) *In vitro* cleavage assay with fluorogenic R247 and R247H peptides. The fluorescence signal from peptides was monitored at the indicated time in different concentrations of trypsin (C) and rT11D (D). The values in the graphs are expressed as the means ± SDs from triplicate samples.

VP4 is a major component of the outer layer of the RVA virion. Proteolytic cleavage of VP4 into VP5* and VP8* is induced by exogenous trypsin, and this activation process is a prerequisite for the infection of permissive cells (27). To investigate the mechanism of the trypsin-independent growth of RVAs in TTSP-expressing cells, we examined whether TMPRSS11D mediates the proteolytic cleavage of VP4. Immature RVA virions carrying uncleaved VP4 were harvested from MA104 cells infected with an RVA in the absence of trypsin (30). The virions were incubated with different recombinant TMPRSS11D (rT11D) concentrations. *In vitro* cleavage of VP4 was evaluated by Western blotting using an anti-VP5* antibody that recognizes both uncleaved VP4 (88 kDa) and cleaved VP5* (60 kDa) (31). Proteolytic cleavage of VP4 was detected and enhanced in an rT11D concentration-dependent manner (Fig. 5A).

For inferring the cleavage recognition site of TMPRSS11D, we utilized a biochemical peptide cleavage assay that uses fluorescence resonance energy transfer (FRET) peptides, which were used for evaluating the viral structural protein cleavage caused by proteases (32, 33). VP4 possesses three conserved arginine residues at the trypsin-targeted site, and the arginine residue at position 247 (R247) is essential for VP4 cleavage and activation (27, 34). We therefore designed a peptide substrate corresponding to the amino acid sequence between 242 and 251 of SA11 VP4 (R247 peptide: DVIHYRAQAN) and labeled it with a FRET pair of the fluorophore EDANS and quencher DABCYL. Peptide cleavage terminates FRET quenching and emits a fluorescence signal from EDANS (Fig. 5B). To assess the impact of R247 on the peptide cleavage, we designed another FRET peptide (R247H peptide: DVIHYHAQAN) in which R247 of the peptide was replaced by histidine. As expected, the R247 peptide emitted a fluorescence signal, and the signal of the R247H peptide was lower than that of the R247 peptide in the presence of trypsin (Fig. 5C). Similarly, incubation with rT11D induced a fluorescence signal from the R247 peptide but not from the R247H peptide (Fig. 5D). These results suggest that TMPRSS11D and trypsin contribute to proteolytic cleavage in the arginine residue at position 247 of RVA VP4.

### Involvement of TTSPs in the virion assembly and budding steps of RVA infection.

**FIG 6:**
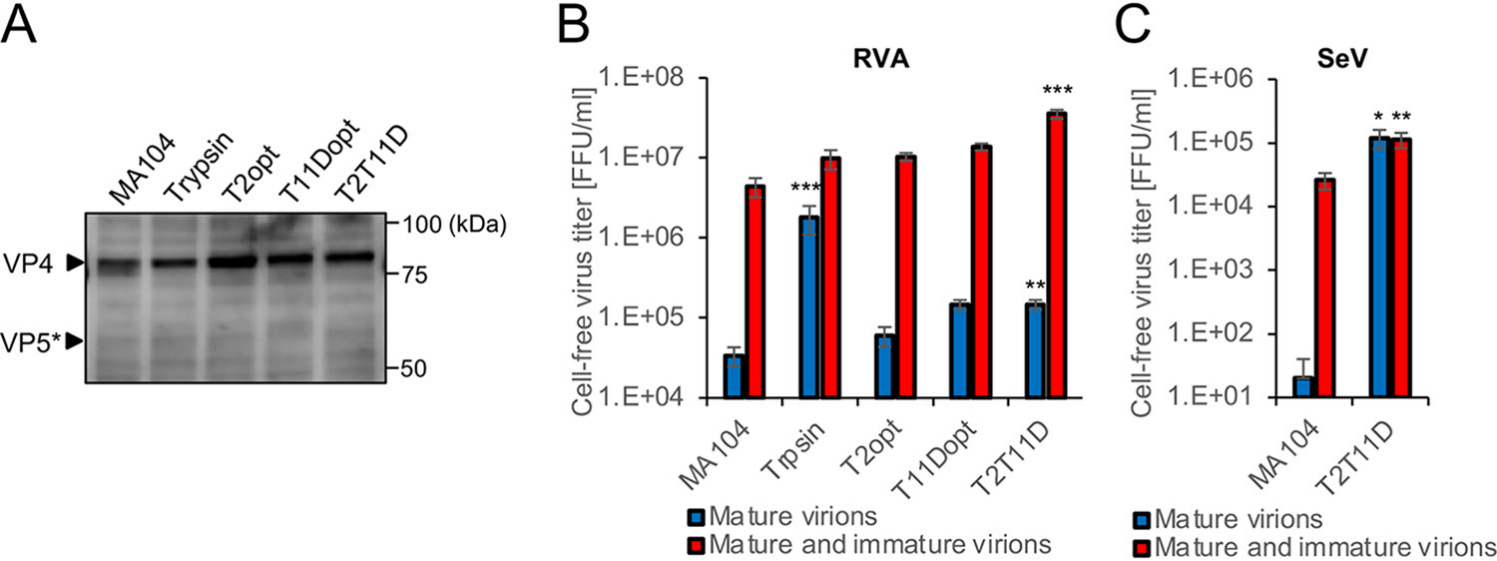
Examination of nascent VP4 cleavage in cells and progeny virions. (A) At 20 h postinfection by the group A rotavirus SA11 strain, cells were lysed and analyzed by Western blotting. Top and bottom arrowheads indicate the signals of uncleaved VP4 and cleaved VP5*, respectively. Progeny virus titers of culture supernatants from cells infected with SA11 (B) and Sendai virus (SeV) (C). Blue bars indicate the titers of mature virions without trypsin pretreatment. Red bars indicate the titers of mature and immature virions with trypsin pretreatment. The values in the graphs are expressed as the means ± SDs from triplicate samples. *, *P* < 0.05; **, *P* < 0.01; ***, *P* < 0.001 by one-way analysis of variance with Dunnett’s test (B) or two-tailed Student's *t* test (C).

It has been reported that both TMPRSS2 and TMPRSS11D cleave the nascent hemagglutinin (HA) protein of influenza virus, and TMPRSS2 cleaves the F protein of paramyxoviruses in cells. Thus, these TTSPs mediate the proteolytic activation of viral proteins during virion assembly and budding (2, 3, 8). We investigated whether proteolytic cleavage of nascent VP4 occurs in T2T11D cells infected with RVA. Western blotting revealed that uncleaved VP4 was the dominant form in RVA-infected cells, whereas VP5* was not detected (Fig. 6A). We also titrated progeny RVA virions harvested from MA104 and TTSP-expressing cells in the culture medium. Samples of progeny RVAs were subjected to a focus assay without trypsin pretreatment to detect infectious virions carrying VP5* (mature virion), and the remaining samples were pretreated with trypsin to detect noninfectious virions (immature virion) carrying VP4 in addition to mature virions. The focus assay demonstrated that infectious mature virions comprised only 2.1%, 1.2%, 1.3%, and 1.2% of progeny viruses from MA104, T2opt, T11Dopt, and T2T11D cells, respectively (Fig. 6B). In contrast with the findings for RVAs, Sendai virus (SeV), a representative paramyxovirus strain, produced matured and infectious progeny virions in T2T11D cells without trypsin supplementation (Fig. 6C), consistent with a previous report (8). These results suggested that TMPRSS2 and TMPRSS11D are unlikely to activate RVA virions during progeny virus production.

### Involvement of TTSPs in the viral entry step of RVA infection.

**FIG 7:**
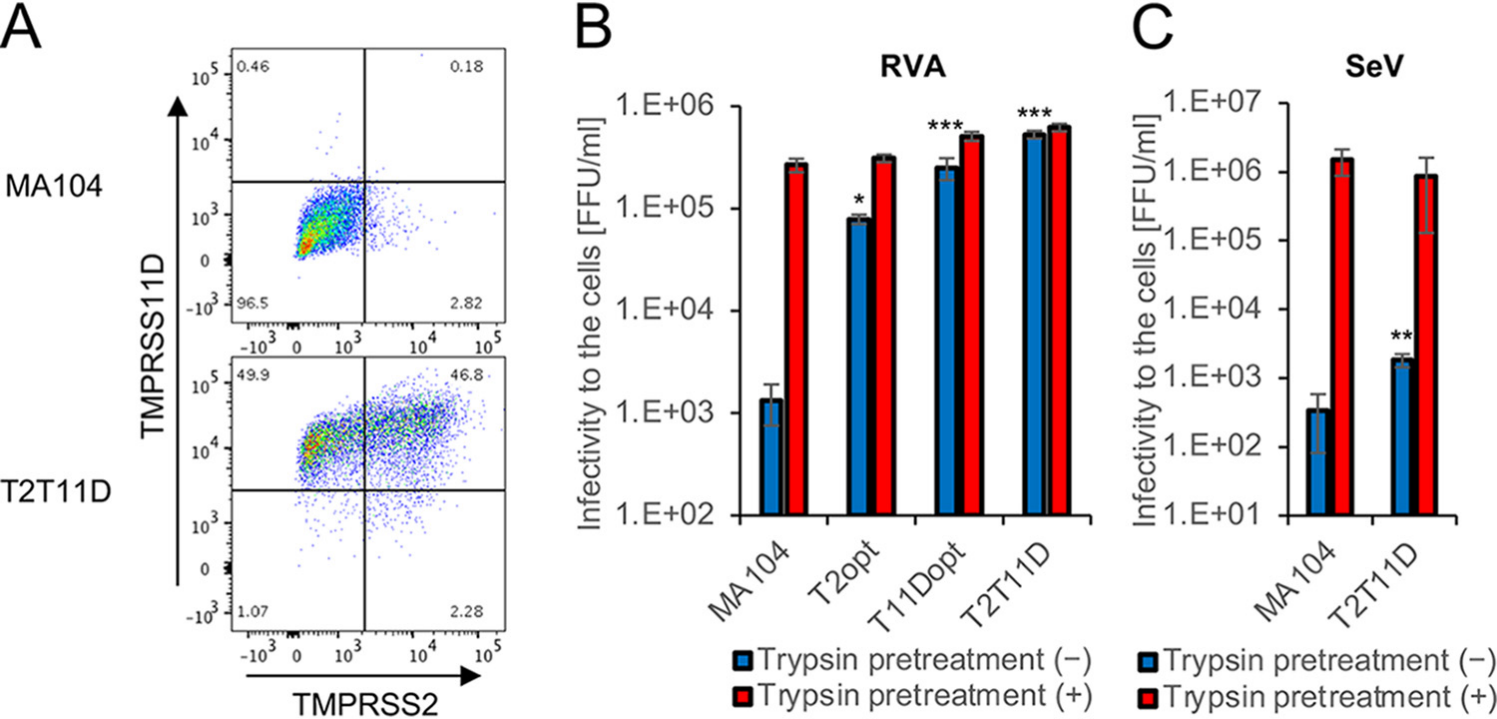
Proteolytic activation of group A rotaviruses (RVAs) on the surface of type II transmembrane serine protease (TTSP)-expressing cells. (A) Cell surface expression levels of TMPRSS2 and TMPRSS11D in MA104 and T2T11D cells. Nonpermeabilized cells were double-stained with anti-TMPRSS2 and anti-TMPRSS11D antibodies and analyzed via flow cytometry. Infectivity of immature virions in TTSP-expressing cells. Immature virions were harvested from MA104 cells infected with the RVA Wa strain (B) or Vero cells infected with the Sendai virus (SeV) (C) in the absence of trypsin. A portion of immature virions was activated by trypsin pretreatment. The infectivity of immature (blue bars) and mature (red bars) virions was determined using a focus assay. The values in the graphs are expressed as the means ± SDs from triplicate samples. *, *P* < 0.05; **, *P* < 0.01; ***, *P* < 0.001 by one-way analysis of variance with Dunnett’s test (B) or two-tailed Student's *t* test (C).

It has been reported that both TMPRSS2 and TMPRSS11D cleave the viral S protein and facilitate the infectious entry of immature coronaviruses (4, 9). In addition, TMPRSS11D has also been reported to mediate the proteolytic activation of viral HA and subsequent entry of immature influenza viruses (3). We next asked whether TMPRSS2 and TMPRSS11D facilitate the infectious entry of immature RVA virions. Flow cytometric analysis revealed the dual expression of TMPRSS2 and TMPRSS11D on the surfaces of nonpermeabilized T2T11D cells, which was consistent with previous findings (3), whereas heterogeneous expression of TMPRSS2 was observed in T2T11D cells (Fig. 7A).

Immature RVA virions were harvested from MA104 cells infected with Wa in the absence of trypsin. The immature virions had low infectivity in MA104 cells, and trypsin treatment for RVA activation rendered the virions highly infectious (Fig. 7B). Notably, TTSP-expressing cells were much more susceptible to infection by the immature virions (Fig. 7B). In particular, T2T11D cells exhibited the highest susceptibility to RVA infection and permitted the infectious entry of approximately 85% of immature virions (Fig. 7B). In contrast to the results for RVAs, T2T11D cells were resistant to infection by immature SeV virions (Fig. 7C), consistent with previous findings that TMPRSS2 has no ability to activate immature SeV virions at the entry step (8).

**FIG 8:**
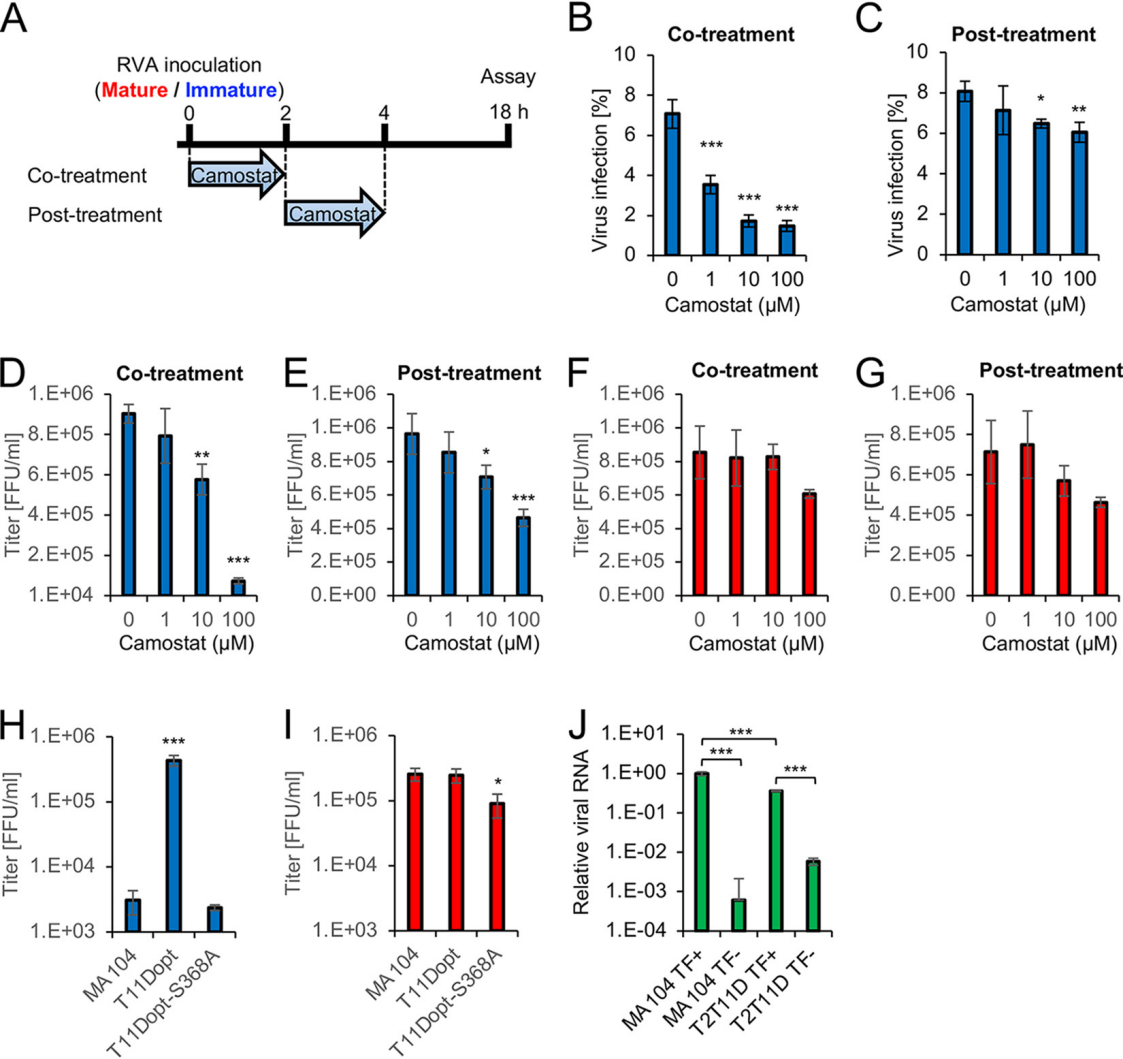
Time of the addition assay of a type II transmembrane serine protease inhibitor. (A to G) Time of the addition assay of a serine protease inhibitor, camostat. (A) Experimental protocol of the time of addition assay. T2T11D cells were treated with camostat for 2 h during (cotreatment) or after (posttreatment) inoculation of group A rotavirus (RVA). (B and C) T2T11D cells infected with immature virions were detected via an indirect immunofluorescence assay. Infection rates were determined via imaging cytometry. T2T11D cells were infected with immature virions (D and E) or mature virions (F and G) with cotreatment (D and F) or posttreatment (E and G) of camostat. Viral titers in the cultures were determined using a focus assay at 18 h postinfection. The indicated cells were infected with immature virions (H) or mature virions (I). Viral titers in the cultures were determined using a focus assay. (J) MA104 and T2T11D cells were inoculated with a mixture of double-layered particles of RVA and transfection reagent (TF+) or double-layered particles only (TF−). The relative levels of viral RNA in cells were measured via qRT-PCR at 16 h posttransfection. The β-actin mRNA levels were used as the reference control. The values in the graphs are expressed as the means ± SDs from triplicate samples. *, *P* < 0.05; **, *P* < 0.01; ***, *P* < 0.001 by one-way analysis of variance with Dunnett’s test (B to I) or one-way analysis of variance with Tukey’s test (J).

We next examined the effect of camostat, a TTSP inhibitor, on the entry of immature RVA virions. T2T11D cells were treated with camostat during RVA virion inoculation (cotreatment) or after exposure of cells to RVA virions (posttreatment) (Fig. 8A). This time of addition assay indicated that cotreatment with camostat had a considerable inhibitory effect on immature RVA infection and the progeny virus titer from T2T11D cells in a concentration-dependent manner, whereas postentry treatment with camostat exerted a less inhibitory effect on immature RVA infection (Fig. 8B to E). In contrast to that for immature RVA infections, mature RVA infections were not inhibited by both cotreatment and posttreatment with camostat, which indicates that the inhibitor does not affect RVA entry except for cleavage of VP4 (Fig. 8F and G). In addition, we examined the infectivity of immature and mature RVAs in T11Dopt cells and T11Dopt-S368A cells expressing the protease-inactive mutant TMPRSS11D-S368A. During infection by immature RVA, the progeny virus titer was increased by the expression of TMPRSS11D but not by that of the TMPRSS11D-S368A mutant (Fig. 8H). No enhancement of the progeny virus titer was observed in T11Dopt and T11Dopt-S368A cells infected with mature RVA (Fig. 8I). Transfection of cells with double-layered particles (DLPs), which are RVA particles that lack an outer membrane, bypasses the viral entry step and establishes infection (35, 36). To further determine the effect of TTSPs on the process of RVA infection, we transfected DLPs into cells and analyzed the level of viral RNA at 16 h posttransfection. The viral RNA level in DLP-transfected T2T11D cells was lower than that in DLP-transfected MA104 cells (Fig. 8J), which indicates that the expressions of TMPRSS2 and TMPRSS11D had no positive effect on postentry stages of RVA infection. Taken together, these results indicated that both TMPRSS2 and TMPRSS11D facilitate the infectious entry of immature RVAs through their protease activity.

## DISCUSSION

In this study, we investigated the role of host TTSPs in RVA infection and spread. Based on the observations in this study, we propose a model in which TMPRSS2 and TMPRSS11D mediate proteolytic activation at the viral entry step and facilitate trypsin-independent infection by RVAs in MA104 cells. First, TMPRSS2 and TMPRSS11D expression facilitated trypsin-independent infection and progeny virus production. Second, the enhancement of RVA infection by TMPRSS2 and TMPRSS11D was dependent on their protease activity. Third, TMPRSS11D induced proteolytic cleavage of the immature form of the viral spike protein VP4. Fourth, cells expressing TMPRSS2 and TMPRSS11D permitted the infectious entry of immature RVA virions, and this infection was inhibited by treatment with a protease inhibitor. In studies of other viruses, similar observations were considered evidence of TTSP-dependent infection (2–12).

It has been reported that TMPRSS2 activates severe acute respiratory syndrome (SARS) coronavirus at the entry step but not during virion assembly (4). Conversely, influenza viruses and SeV have been reported to be activated by TMPRSS2 during progeny viral production and release (3, 8). Our work indicated that immature RVA virions were dominant in the progeny viruses from TTSP-expressing cells and MA104 cells, and proteolytic cleavage of nascent VP4 was not observed in RVA-infected cells. Thus, both TMPRSS2 and TMPRSS11D are likely to activate immature RVAs, specifically, during the entry process. Characterization of the proteolytic cleavage of VP4 on the cell surface is required to further understand the mode of RVA activation by TTSPs. Importantly, in contrast to the findings for RVAs, T2T11D cells activate SeV virions during progeny virus production but not incoming SeV virions in the early phase of infection (8), indicating that the process of virus activation of TTSPs is virus specific but not cell specific.

RVA VP4 exhibits extensive sequence divergence, and all recognized VP4 genes are currently classified into 51 genotypes (P[1] to P[51]) based on their nucleotide sequences by the Rotavirus Classification Working Group (37). In this study, we confirmed the trypsin-independent growth of RVAs in TTSP-expressing cells using laboratory and clinical strains with different VP4 genotypes, including SA11 (genotype P[2]), Wa (genotype P[8]), DS-1 (genotype P[4]), and Azuk-1 (genotype P[29]) (37, 38). These results suggest that TMPRSS2 and TMPRSS11D mediate the proteolytic activation of various RVA strains, including clinical isolates.

T2T11D cells were highly susceptible to infection by immature RVA virions, and their infectivity was similar to that of mature RVA virions following trypsin activation. Therefore, T2T11D cells could be applicable to titration assays of RVAs without trypsin activation. Moreover, RVA growth in T2T11D cells without trypsin supplementation was equal to or higher than that in MA104 cells in trypsin-supplemented culture. This advantage would assist in RVA assays. Indeed, an approach to increasing RVA growth has led to the reverse genetic rescue of a human RVA strain with a low replication rate (39). The use of T2T11D cells or TTSP-transduced cells would also represent a practical approach to the large-scale manufacture of high-yield live rotavirus vaccines without any protease supplements, such as porcine trypsin, which is considered the main source of porcine circovirus contamination in vaccine products (40–43).

The growth of RVA in T2T11D cells was observed but impaired in the presence of high FBS concentrations, which indicates that FBS supplementation neutralizes the protease activity of TTSPs and trypsin. However, we could not exclude the possibility of the contamination of neutralizing antibody against RVA in FBS (44).

Previous studies mainly targeted respiratory viruses for the investigation of trypsin-independent infection by host TTSPs, with the exception of porcine endemic diarrhea virus (5, 12). Our work revealed the association between TTSPs and RVAs, which are representative enteric virus species, extending the study of TTSP-mediated viral infection to enteric viruses. Because RVA infection occurs in the gut in the presence of trypsin, the question of whether TTSPs are involved in RVA infection *in vivo* has yet to be elucidated. Because TMPRSS2 and TMPRSS11D are expressed in enterocytes lining the villi of the small intestine, which are targets of RVA infection (13, 45, 46), *in vivo* analysis with TMPRSS2 and TMPRSS11D knockout animal models will be an important experiment in future studies (16–20).

## MATERIALS AND METHODS

### Cells and viruses.

Monkey kidney MA104 cells were maintained in Eagle’s minimum essential medium (MEM) supplemented with 10% FBS (Gibco; Thermo Fisher Scientific, Waltham, MA) and 10% tryptose phosphate broth (TPB). Monkey kidney Vero cells were maintained in Dulbecco’s modified Eagle’s medium (DMEM) supplemented with 10% FBS. Sendai virus (VR-105; ATCC, Manassas, VA) was propagated in Vero cells in serum-free DMEM containing trypsin (0.5 μg/ml) in static culture. Simian RVA SA11 (genotype G3P[2], VR-1565; ATCC) and human RVA Wa (genotype G1P[8], VR-2018; ATCC) strains were propagated in MA104 cells in serum-free MEM containing 10% TPB and trypsin (0.5 μg/ml) in static culture. Human RVA DS-1 (genotype G2P[4], VR-2550; ATCC) and bovine RVA Azuk-1 (genotype G21P[29]) (47) strains were propagated in MA104 cells in serum-free MEM containing 10% TPB and trypsin (0.5 μg/ml) in roller bottles (Corning, Corning, NY).

### Generation of TTSP-expressing MA104 cells.

The human TMPRSS2, human TMPRSS11D, human TMPRSS11E, and human TMPRSS13 genes were individually cloned into the self-inactivating lentiviral vector plasmid CSII-CMV-MCS-IRES2-Bsd (48), which was kindly provided by H. Miyoshi (RIKEN BRC, Tsukuba, Japan). Codon-optimized TMPRSS2 and TMPRSS11D genes were designed by the IDT codon optimization tool (Integrated DNA Technologies, Coralville, IA), were synthesized as custom-made DNA fragments (gBlocks; Integrated DNA Technologies), and were cloned into CSII-CMV-MCS-IRES2-Bsd and pLVSIN-CMV Pur (TaKaRa Bio, Kusatsu, Japan), respectively. For the preparation of lentivirus particles, the lentiviral vector plasmid and lentiviral high titer packaging mix (TaKaRa Bio) were cotransfected into Lenti-X 293T cells (TaKaRa Bio) with TransIT-293 transfection reagent (Mirus Bio, Madison, WI). The culture supernatant containing lentiviral vectors was filtered through a Minisart 0.45-μm-pore size filter (Sartorius, Göttingen, Germany) and then inoculated to MA104 cells in the presence of 10 μg/ml Polybrene. To generate T2T11D cells, MA104 cells were inoculated with lentiviral vectors expressing TMPRSS2 and TMPRSS11D and were selected with blasticidin S (for TMPRSS2-expressing cells) and puromycin (for TMPRSS11D-expressing cells).

### Western blotting.

Cells were lysed in lysis buffer (1% NP-40, 20 mM Tris-HCl [pH 7.5], 150 mM NaCl, 5 mM EDTA) supplemented with cOmplete ULTRA protease inhibitor cocktail (Roche Diagnostics, Mannheim, Germany). Lysate samples were resolved by SDS-PAGE and transferred onto Immobilon-P polyvinylidene difluoride (PVDF) membranes (Merck, Burlington, MA). Blots were incubated with the following primary antibodies: anti-TMPRSS2 (ab92323; Abcam, Cambridge, UK) antibody diluted with 5% skim milk in TBST (25 mM Tris-HCl [pH 7.5], 137 mM NaCl, 2.7 mM KCl) and anti-TMPRSS11D (GTX117370; GeneTex, Irvine, CA), anti-TMPRSS11E (PA5-48775; Invitrogen, Thermo Fisher Scientific), and anti-TMPRSS13 (GTX117425; GeneTex) antibodies diluted with Signal Booster (Beacle, Kyoto, Japan). Horseradish peroxidase (HRP)-conjugated anti-β-actin antibody (PM053-7; MBL, Nagoya, Japan) was used as a loading control. Immune complexes were detected using HRP-conjugated secondary antibodies and Immobilon Western chemiluminescent HRP substrate (Merck).

### Multicycle growth of RVAs in TTSP-expressing MA104 cells.

Cells were infected with SA11 at a multiplicity of infection (MOI) of 0.01 or with Wa, DS-1, or Azuk-1 at an MOI of 0.1. After 1 h of incubation, cells were washed with phosphate-buffered saline (PBS) and cultured in serum-free MEM with 10% TPB, with or without 0.5 μg/ml trypsin. In Fig. 4, cells were cultured in MEM supplemented with 10% TPB and the indicated concentration of FBS. Cells infected with the SA11 or Wa strain were grown under static culture, but cells infected with DS-1 or Azuk-1 were grown under roller culture conditions using tissue culture tubes (91243; TPP, Trasadingen, Switzerland) for efficient virus propagation. At 48 hpi, RVA infection and growth were evaluated by immunofluorescence, titration, and qRT-PCR assays.

### Indirect immunofluorescence assay.

Cells infected with RVA or SeV virions were fixed with 3.7% buffered formaldehyde, permeabilized with ice-cold methanol, and incubated with anti-RVA polyclonal antibody (AB1129; Merck), anti-RVA VP6 monoclonal antibody P3-1 (49), or anti-SeV polyclonal antibody (PD029; MBL). Alexa Fluor 488-conjugated anti-goat, anti-mouse, or anti-rabbit IgG antibodies (Invitrogen) were used as secondary antibodies. Nuclei were stained with Hoechst 33342 (Invitrogen). Fluorescence images were captured using a fluorescence microscope (IX73; Olympus, Tokyo, Japan).

### qRT-PCR assay.

Total RNA was extracted from RVA-infected cells and reverse transcribed into cDNA using a PureLink RNA minikit and SuperScript IV VILO master mix (Invitrogen). The cDNA was subjected to qPCR analysis using Thunderbird probe qPCR mix (TOYOBO, Osaka, Japan). The primer and probe sequences were as follows: SA11 *VP6*, 5′-AACCCGCTCATGATAATTTGATGG-3′ (nucleotide positions 532 to 555 in GenBank LC178568), 5′-GCGTTAATAGCACATGAGTAGTCAAA-3′ (nucleotide positions 600 to 625), and 5′-6-carboxyfluorescein (FAM)-CCAGCGACTTGAATTTCCGATCCTGCGTT-black hole quencher 1 (BHQ1)-3′ (nucleotide positions 570 to 598); Azuk-1 *VP6*, 5′-TCCAGTTGATGAGACCACCAAA-3′ (nucleotide positions 875 to 896 in GenBank LC178568), 5′-GCATGATGTTCAAATGGCTGTG-3′ (nucleotide positions 932 to 953), and 5′-FAM-TGACACCAGCAGTAGCAGCACTATTTCCGA-BHQ1-3′ (nucleotide positions 899 to 928). The amplicon sizes of SA11 *VP6* and Azuk-1 *VP6* are 94 bp and 79 bp, respectively. The primer and probe sequences for human RVA *NSP3* and nonhuman primate β-actin were described previously (50, 51).

### Flow cytometry.

Cells were washed with PBS and detached from the culture dishes using nonenzymatic cell dissociation buffer (Gibco). The cells were incubated with a mixture of anti-TMPRSS2 rabbit polyclonal antibody (A1979; ABclonal, Tokyo, Japan) and anti-TMPRSS11D mouse polyclonal antibody (H00009407-B01; Abnova, Taipei, Taiwan) in PBS supplemented with 1% bovine serum albumin (BSA) at 4°C for 30 min and were then stained with Alexa Fluor Plus 488-conjugated anti-rabbit IgG and Alexa Fluor Plus 647-conjugated anti-mouse antibodies (Invitrogen). Data were collected using the fluorescence-activated cell sorter (FACS) Canto system (BD Biosciences, San Jose, CA) and analyzed using the FlowJo software (BD Biosciences).

### Virus infection assay with TTSP inhibitors.

To evaluate the effect of TTSP inhibitors on the multicycle growth of RVAs, the Wa strain was inoculated into cells at an MOI of 0.1 for 1 h. Cells were washed with PBS and then cultured in MEM supplemented with 10% TPB and the TTSP inhibitors AEBSF (A8456; Sigma-Aldrich, St. Louis, MO), leupeptin (L2884; Sigma-Aldrich), and camostat (039-17761; Wako, Osaka, Japan). At 48 hpi, total RNA was extracted from the cells, and qRT-PCR assays were performed as described above. To evaluate the effect of TTSP inhibition on viral entry, cells were treated with camostat during (cotreatment) or after (postentry treatment) virus inoculation. At 16 hpi, RVA-infected cells were subjected to an immunofluorescence assay, as described above. Infection rates were calculated as the proportion of RVA-positive cells among all cells via imaging cytometry using IN Cell Analyzer 2000 and IN Cell Investigator software (GE Healthcare, Chicago, IL).

### *In vitro* cleavage assay of RVA VP4.

To prepare immature RVA virions carrying uncleaved VP4, cells were infected with the SA11 strain in MEM supplemented with 10% TPB and 5% FBS, but without trypsin. The culture was subjected to freeze-thaw cycles and passed through a 0.45-μm filter. The virions in the culture were ultracentrifuged using an SW32Ti rotor (Beckman Coulter, Brea, CA) for 2 h at 133,900 × *g*, and the pellet was resuspended in PBS. The immature RVA virions were incubated with recombinant human TMPRSS11D (2695-SE; R&D Systems, Minneapolis, MN) in assay buffer (50 mM Tris-HCl [pH 9.0], 0.05% Brij-35) or trypsin in PBS at 37°C for 3 h. VP4 cleavage was detected via Western blotting using an anti-VP5* antibody kindly provided by S. Komoto (Fujita Health University School of Medicine, Japan) (31).

### Biochemical peptide cleavage assay.

Internally quenched fluorogenic peptides with DABCYL/Glu (EDANS) modification were custom-synthesized by GenScript (Piscataway, NJ). The peptides (50 μM) were mixed with different concentrations of trypsin in PBS or recombinant human TMPRSS11D in the assay buffer (50 mM Tris-HCl [pH 9.0], 0.05% Brij-35) on one-half area OptiPlate-96 F (PerkinElmer, Waltham, MA). The fluorescence signal was measured every 2 min on an EnVision microplate reader (PerkinElmer).

### Titration by focus assay.

For titration, RVA virions were harvested from cells via freeze-thaw cycles and activated with 20 μg/ml trypsin. Monolayers of MA104 cells were incubated with the harvested RVA samples for 1 h and cultured for 16 h in the overlay medium (MEM supplemented with 2% FBS and 0.5% methyl cellulose). The virus titer (calculated as focus forming unit [FFU] per ml) was determined using an immunofluorescence assay, as described above. SeV virions were titrated via a focus assay using Vero cells. To quantify the infectivity of immature virions in TTSP-expressing cells, immature virions of the RVA Wa strain and SeV were prepared by culture in trypsin-free medium. A portion of the immature virions was activated by pretreatment with trypsin (20 μg/ml) for 30 min. The infectivity of the mature and immature virions in TTSP-expressing cells was determined using a focus assay.

### Double-layered particle transfection.

A DLP was prepared as described previously (52). Briefly, RVA SA11 virions (triple-layered particles) in the culture supernatant were incubated with 10 mM EDTA for 20 min for conversion to DLPs and then pelleted by ultracentrifugation using an SW32Ti rotor for 2 h at 133,900 × *g*. The pellet was resuspended in TBS with 10 mM EDTA, and 2.2 g of CsCl was added to 4.4 ml of the DLP suspension. The mixture was ultracentrifuged using an SW60Ti rotor for 20 h at 259,000 × *g*. DLPs by banding on the CsCl gradient were collected, pelleted by ultracentrifugation, and resuspended in PBS. To introduce DLPs into cells, DLPs were incubated with Lipofectamine 2000 in Opti-MEM (Invitrogen) for 20 min and then inoculated into cells. Sixteen hours posttransfection, total RNA was extracted from cells and analyzed by qRT-PCR assay as described above.

### Statistical analysis.

A two-tailed Student's *t* test was used to determine statistical significance in Fig. 6C and 7C. One-way analysis of variance was employed to determine statistical significance in other figures.
